# Materials library collections as tools for interdisciplinary research

**DOI:** 10.1080/03080188.2018.1435450

**Published:** 2018-03-08

**Authors:** S. E. Wilkes, M. A. Miodownik

**Affiliations:** Institute of Making, University College London, London, UK

**Keywords:** Materials libraries, interdisciplinarity, materials research, social science, design research

## Abstract

This paper examines how materials libraries are used as tools for interdisciplinary collaboration in 3 research projects that inhabit a disciplinary triangle between materials research, design and user needs: *PhysFeel*, which explores how materials collections can be used in psychological therapies; *Light.Touch.Matters*, a design-led project to develop new smart materials; and *Hands of X*, which uses materials collections to develop a bespoke prosthetics service. The paper analyses and contrasts these case studies to better understand the affordances and limitations of materials collections when used as research, translational and design tools. We conclude that in collaborations between materials researchers, designers and end users, tensions arise as a result of the primacy that each partner gives to creativity, the development of new knowledge and to solving societal problems. The use of a materials library addresses many of these issues but is not a panacea for all the problems associated with interdisciplinary working.

## 1. Introduction

**Figure 1.**
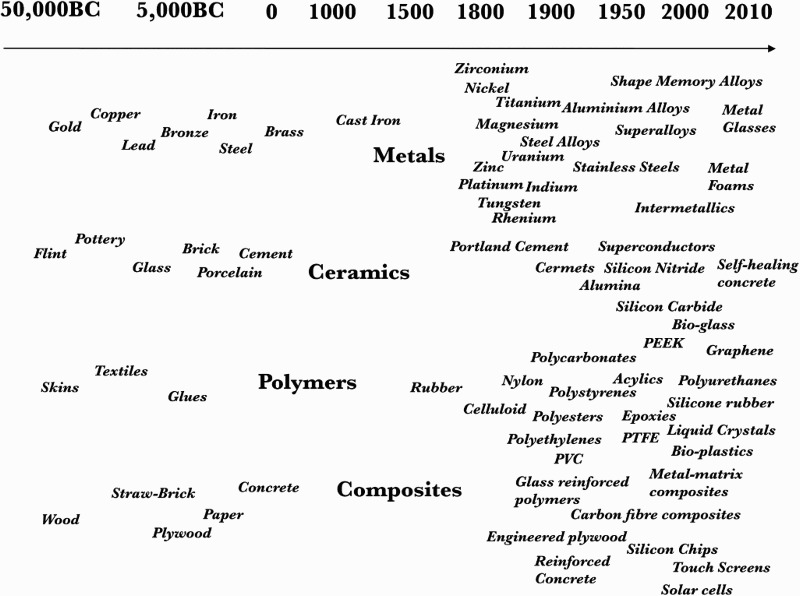
Schematic of the history of materials discovery, adapted from Ashby, Shercliff, and Cebon (2013).

Everything is made from something, and throughout history, the materials from which we make things have become ever more diverse and complex. Figure 1 shows a schematic of the growth of new materials that has occurred in the last 50,000 years, much of which is concentrated in the period from the end of the nineteenth century. For makers and manufacturers, selecting materials has become increasingly difficult because of the vast choice. In 1997, Philip Ball estimated that there were up to 80,000 materials to choose from when fabricating an artifact (Ball 1997); current estimates put the number at 160,000 (Ashby, Shercliff, and Cebon 2013). It is thus becoming increasingly difficult for artists, designers, engineers and architects to have in-depth knowledge across such a broad spectrum of materials.

Materials libraries emerged to address this problem. Like a library of books, these are repositories of knowledge, but instead of books they contain the materials themselves. They provide access to physical samples of materials: many aspects of materials are currently unquantifiable so a hands-on experience is a vital part of navigating the materials selection process. Materials libraries take many forms: commercial businesses, not-for-profit organizations, in-house facilities of commercial companies and those embedded in educational establishments (Laughlin 2010). The unifying principle behind most of these libraries is that they exist as design tools to help architecture, design and engineering practitioners and students to specify materials (Wilkes 2011). This paper focuses on the materials library based at UCL’s Institute of Making (Institute of Making 2017), and the uses to which it has been put.

All materials libraries rely to an extent on the swatch: different materials that take the same form to allow for direct, hands-on comparison of their physical, sensory and aesthetic properties. What distinguishes UCL’s materials library is the form that these swatches take and the uses to which they are put. Moving ‘beyond the swatch’, Laughlin (2010) developed specially made ‘material-object’ sets. These sets took recognizably functional object forms such as cubes, spheres, tuning forks and bells and made them into multiples, each made from different materials in order to explore the relationship between form, function and materiality. These material collections were developed with several aims in mind. As well as serving as a tool to help designers experience and understand materials, they were also conceived as tools to communicate and translate concepts between materials-oriented disciplines; between an artist and engineer or a materials scientist and an anthropologist for example. The tuning fork collection, for instance, allowed users of the library to engage with and understand the factors that affect the acoustic properties of different materials. This led to the development of materials collections as research tools for exploring human experiences of materials from an interdisciplinary perspective, taking into account how their chemical, physical and mechanical properties relate to their sensory and aesthetic properties. Our materials collections are used as the focus for psychophysical experiments where we systematically explore how measurable material properties such as density, stiffness and electrode potential relate to people’s subjective ‘sensoaesthetic’ experiences of their tactile, gustatory, somatosensory and acoustic qualities (Laughlin 2010; Laughlin et al. 2011; Wongsriruksa et al. 2012, Howes et al. 2014; Laughlin and Howes 2014; Wilkes et al. 2016).

Over the last few years, we have been using our materials library as a research tool in a range of collaborations involving, for example, art historians, English literature scholars, historians of science, chemists and clinicians. In this paper, we focus on three collaborative projects that all explored the impact of materials in health and well-being applications but used our materials library in different ways. The first project we discuss is *PhysFeel*, a collaboration between social scientists to understand how materials could be used to communicate affective phenomena: the raw, subjective experiences perceived and cognitively processed as moods, feelings, attitudes and emotions (Scherer 2005). The second case study is *Light.Touch.Matters*, a European consortium of materials researchers and designers that aimed to develop a new smart material. The third example is *Hands of X*, a project in which we worked with design researchers to develop new approaches to prosthetic design.

**Figure 2.**
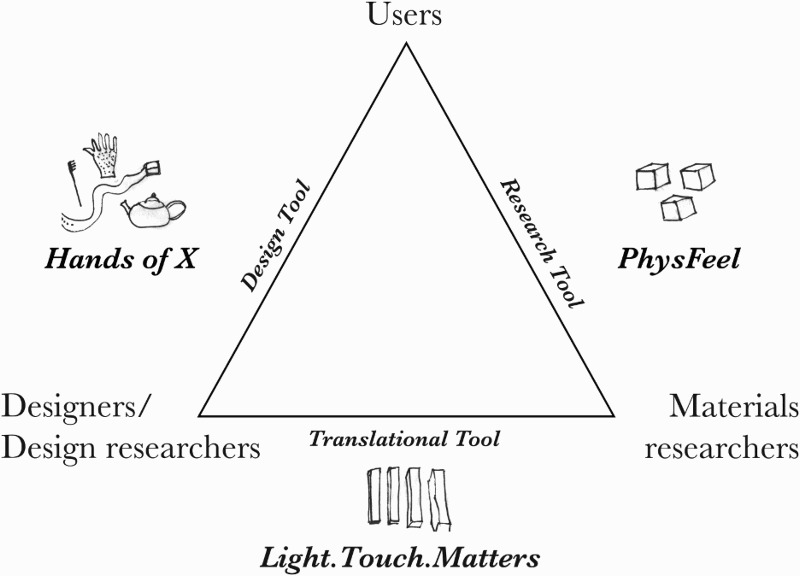
Schematic of the tripartite relationship between the important players in the journey from materials innovation to their application and how they relate to the case studies presented in this paper.

The reason for including the three case studies is to highlight and explore the interdisciplinary relationship between the important players in the journey from the invention of a material to its application: we categorize these players as the materials researchers, the designers or design researchers and the end users. Figure 2 shows a schematic of the tripartite relationship that positions this paper and where our case studies fit with respect to them.^1^

As Fitzgerald and Callard (2015) have noted, literature on interdisciplinarity is often characterized by an ‘arid rhetoric’, with little critical attention paid to the mechanics and methodological impact of these collaborations on researchers. By contrast, this paper attempts to reveal the practicalities of using collections of materials as tools for interdisciplinary research. In the featured case studies, we explore how the materials library enabled three different interdisciplinary groups to resolve translation issues and think through research questions they could not otherwise contemplate. We also propose that materials libraries be thought of as epistemic objects in the research process (Knorr-Cetina 1997; Rheinberger 1997): unlike the technological objects and commodities that are often the ‘finished’ outputs of collaborative materials and design research projects, these collections of materials are always incomplete and in process; they are constantly being remade and reclassified. The materials library sits at the centre of our research process, motivating collaborative work: it is both an agent that brings together different players around a research question and a tool to direct materials from invention to application. However, we recognize that materials libraries should not be seen as a panacea for all the problems and labour of interdisciplinary work. Moving beyond a ‘frictionless imaginary’ (Callard and Fitzgerald 2016), this paper also explores some of the complications and limitations of a materials-oriented approach.

## 2. Case studies

### 2.1. PhysFeel

The initial aim of the *PhysFeel* project was to investigate how our materials library could be used in the context of psychological therapies. Self-report questionnaires are commonly used to measure emotions in order to evaluate the outcomes of therapy or meditation. These questionnaires often list emotion descriptors such as happy, angry, amused and embarrassed and ask people how often or intensely they have experienced those emotions on a numerical scale (for examples see Power 2006; Fredrickson 2013). This study was motivated by reports from clinical practice that filling in questionnaires can be frustrating and repetitive for some mental health service users, leading to participant disengagement (Law and Wolpert 2014). We wanted to explore whether using specially designed material-object sets instead of questionnaires could make the measurement of affect more engaging for participants, whilst still providing good psychometric validity.

This study was informed by research in the area of cognitive linguistics that demonstrates a metaphorical relationship between emotion words such as sadness, anger, fear and love and perceptual dimension labels such as weight, temperature and colour (Lakoff and Kövecses 1987; McMullen and Conway 2002; Hurtienne, Stößel, and Weber 2009; Cavanaugh, MacInnis, and Weiss 2016). For example, Cavanaugh, MacInnis, and Weiss (2016) recently demonstrated that perceptual dimensions such as colour, size, speed, temperature and weight are consistently used to differentiate positive from negative emotions, and high arousal from low arousal emotions. However, in this existing literature, relationships between perceptual dimensions and emotions are only explored verbally: participants are asked to pair emotion words with perceptual dimension descriptors.

This study therefore also explored whether material-object sets could enable a different kind of conversation than that made possible through verbal communication alone. Numerous researchers point to the limitations of using verbal behaviours to obtain a complete picture of a person’s affective well-being (Robins and John 1997; Williams, Davies, and Chadury 2000; Paulhus and Vazire 2009). Inspired by research in the fields of interaction design, design research and sensory science (Karana, Hekkert, and Kandachar 2009; Spence and Gallace 2011; Okamoto, Nagano, and Yamada 2013), our study moved away from using exclusively verbal stimuli and semantic differential methods to capture peoples’ perceptual and affective experiences. Similar developments in psychology have seen a proliferation of non-verbal, measures of affect such as the Facial Affective Scale (McGrath, De Veber, and Hearn 1985) and the Self-Assessment Manikin (Bradley and Lang 1994). As Isbister et al. (2006) have noted, much of this existing work uses two-dimensional and anthropomorphic images or colours to differentiate emotions. This study, on the other hand, explored whether it is possible for people to consistently represent and communicate the experience and intensity of emotions via the look and feel of three-dimensional objects that varied along one material property.

In order to explore how material properties might map onto emotions, we initially chose to make material-object sets that varied along five material dimensions. We chose those material properties that correlated with the metaphors and perceptual dimensions that had been the focus of previous psychological and psychophysical research: temperature (Lakoff and Johnson 1999; Hurtienne, Stößel, and Weber 2009; Waggoner 2010); roughness (Zuo, Hope, and Jones 2013; Cavanaugh, MacInnis, and Weiss 2016); density (McMullen and Conway 2002; Hurtienne, Stößel, and Weber 2009); softness (Cavanaugh, MacInnis, and Weiss 2016) and elasticity (Waggoner and Palermo 1989). We later added a sixth material dimension of transparency or opacity, as explored by Cavanaugh, MacInnis, and Weiss (2016), in order to span the different sensory modalities of touch and sight.

**Figure 3.**
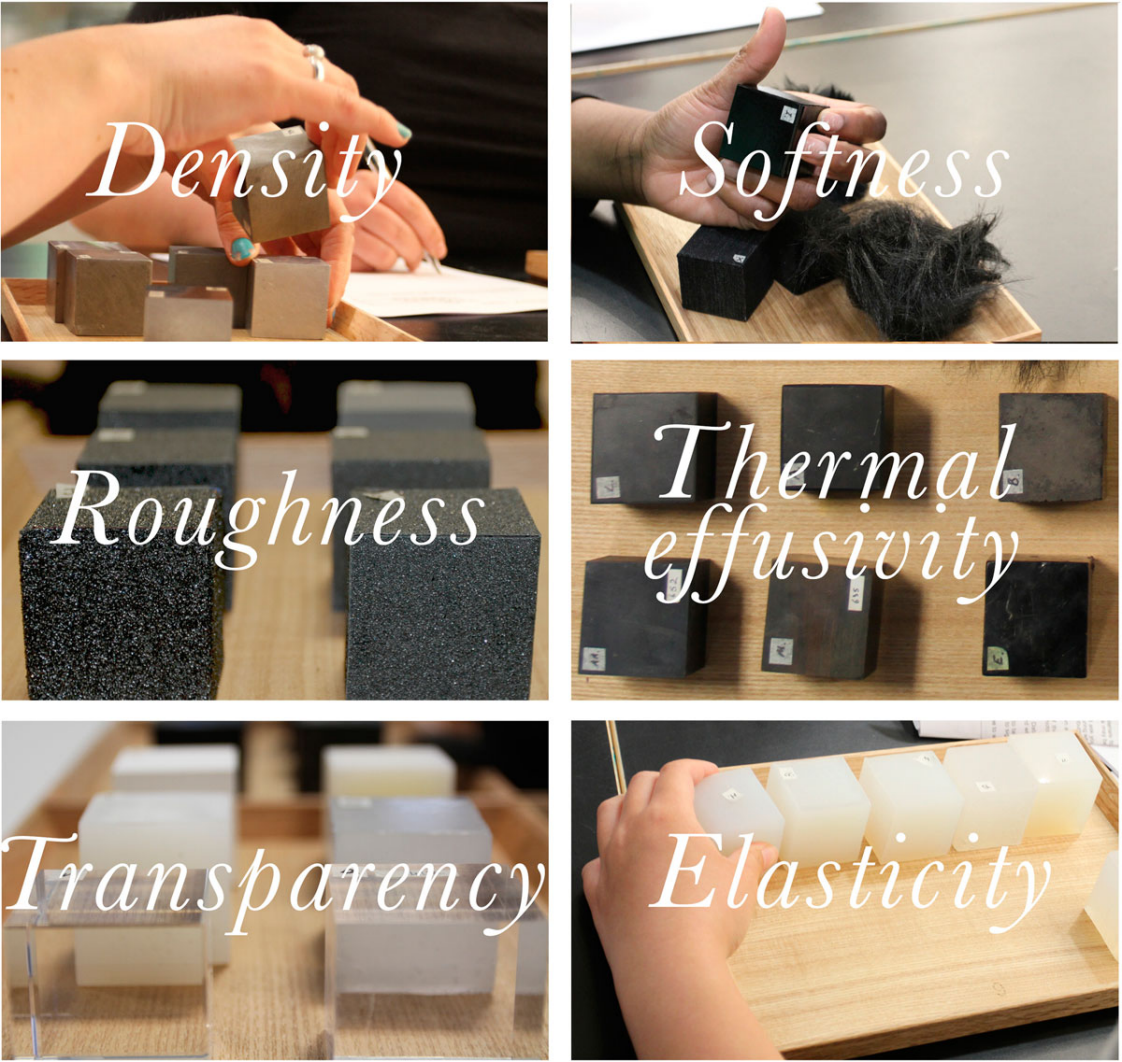
Photographs and property descriptors of our material collection of cubes used in the *PhysFeel* focus groups. Density set: tungsten, solder, cast iron, zinc, titanium and aluminium, selected for their different densities using the Cambridge Engineering Selector (CES Edupack 2016). Softness set: fake fur, fun fur, silk velvet, synthetic velvet, denim and PVC, selected for their different fibre lengths and stiffnesses. Roughness set: different grades of sandpaper from P60 to P800 grit. Thermal effusivity set: concrete, slate, vulcanized rubber, nylon, hardwood and wax, all dyed black, selected for their different thermal properties using the Cambridge Engineering Selector (CES Edupack 2016). Transparency set: plaster of Paris, nylon, polypropylene, polyurethane resin, acrylic and glass, selected for their different optical transparencies. Elasticity set: cast from silicone rubbers of different shore hardnesses (Shore 10 to 50).

We drew on the existing materials library collection to explore what forms and material properties might be useful for communicating emotions. Metal cubes and silicone rubber lollipops, made in the context of other research projects, proved particularly useful for structuring conversations between the research team about the relationship between materials and affect. They inspired the development of a first set of *PhysFeel* prototypes, which took a variety of forms. These prototypes were tested on a pilot focus group with six participants from a range of disciplinary and occupational backgrounds (e.g. computer science, psychology and humanities), and in a range of sighted and unsighted conditions. On the basis of the feedback from this pilot session, we developed a specially made set of 36 material-object cubes (6 collections of 6 cubes), each measuring 40 mm x 40 mm, that varied along the dimensions of elasticity, density, softness, roughness, thermal effusivity and transparency whilst keeping all other properties constant (see Figure 3).

During a series of 6 focus groups, 29 non-clinical participants were then asked to spend 3 minutes handling and looking at each of these cube sets in turn and to write down their responses to a series of questions about their material properties and what emotion they thought could be communicated with the cubes. This exercise was followed by an in-depth group discussion about how they found the task, which was audio recorded. Participants’ written responses detailing the feelings associated with each set were coded and thematically analysed to explore whether the material-object sets were more frequently associated with affective descriptors or non-affective descriptors; whether they were consistently associated with one or more emotion term; and whether they were consistently associated with positive or negative valence. Participants’ verbal responses to the task were also transcribed, coded and subjected to inductive thematic analysis to explore their reasons for assigning emotions to material-object sets as well as their experience of the task.

Since the findings of the study are not the main focus of this article, we will not discuss them in detail here, but as discussed elsewhere (Wilkes et al., forthcoming) there was agreement between participants in assigning emotion terms to the materials collection of cubes. For example, the softness and elasticity cubes were most often associated with happiness; the thermal cubes with sadness and the roughness cubes with anger-irritation. In this paper, our interest is in the role that the materials library played as a research tool, as a translational tool, and as a design tool in this specific context.

The materials library played a crucial role in bringing the research team together, inspiring the research question, structuring initial interdisciplinary conversations about the relationship between materials and affect and influencing the development of the *PhysFeel* materials collection. This physical collection of objects was also absolutely essential for structuring conversations with research participants. However, in the context of this project, the materials library collection was not needed as a translational tool. Collaboration between members of the *PhysFeel* team was relatively frictionless, perhaps because all three researchers came from social science backgrounds. Communication issues only arose when disseminating the findings of this study to the design community.

In the fields of design research, sensory science and consumer psychology there remains an enduring interest in understanding how material properties elicit emotional responses (Norman 2004), contribute to product personality (Ashby and Johnson 2002) and ultimately how designers can use materials to create ‘intended meanings’ with their products (Karana, Hekkert, and Kandachar 2009; Spence and Gallace 2011). Some of the engineering and design practitioners who were interested in the project wanted to use the *PhysFeel* materials collection and associated data to ‘define the emotional qualities of materials’, produce a glossary of material property correlates with emotions and apply it to the manufacture of office equipment, for example. This kind of a semiotic approach to material properties, which treats them as signs that can be transmitted to the user, has validity in design and engineering disciplines (Schütte 2005). However, our approach to the project was influenced by a shared interest in cognitive science and material culture approaches, where there has been a push-back against the idea that materials and artefacts somehow encode memories, thoughts or emotions (Ingold 2000; Malafouris 2004). *PhysFeel* paid close attention to the subjective material and emotional experiences of participants using the sets as prototype tools for psychological therapies. Thus, we felt that transposing the emotional associations that the *PhysFeel* cubes were given by the focus group to materials used in other everyday objects was not straightforward.

Controlled research in a laboratory setting that isolates one measurable material property in order to explore how it relates to subjective experience allows for a direct focus on materiality in a way that a lot of observational research does not. However, we take the stance that the emotional associations of a material cannot be fully understood in isolation from the cultural totality of the whole material in its context of use. As anthropologist Webb Keane comments, individual material qualities ‘must be embedded in something in particular’ and as soon as they are, ‘they are … bound up with other qualities’ (2005). Keane gives the example of redness in an apple, which is bound up with its ‘spherical shape, light weight, sweet flavour, a tendency to rot, and so forth’. He could equally be talking about the properties of glass, silicone rubber or stainless steel, where one physical property like strength is also always bound up with other distinctive physical properties (e.g. gloss, thermal properties and surface roughness) as well as the cultural associations that are a result of the way a material has historically been used. It is for that reason that our materials collections often take a recognizable functional object form to allow us to explore how materials choices impact on specific experiences like eating or wearing a prosthetic hand.

Aside from the question of whether the findings from the *PhysFeel* study could be transposed to other contexts of use, this encounter also raised ethical questions of how the findings might be used by engineering and design practitioners. In the context of *PhysFeel*, we were interested in how material-object sets might induce a positive affective experience and encourage participant engagement in the specific context of mental health interventions (Wilkes et al., forthcoming). However, affective engineering is promoted by some of its proponents as a way of designing products ‘in a way that will evoke a positive impact and make the consumer buy the same product again – in other words creating desire’ (Dahlgaard et al. 2008). As Verbeek (2011) notes, all materials and technologies give shape to what we do, influence how we experience the world and encourage particular kinds of human behaviour, and thus have moral implications. However, when design collides with sales and marketing this raises a particular set of ethical questions about ‘intentionality’ and affective manipulation (Verbeek 2008), discussions of which are rarely seen in the engineering literature.

One of the main aims of the *PhysFeel* project was to develop a tool for psychological therapies, but it did so without the direct involvement of designers, as Figure 2 depicts. This led to friction around the ways the research results generated from one disciplinary perspective are used by researcher and practitioners working from another perspective. Nevertheless, including designers in the team does not indemnify projects against mutual misunderstandings, as we shall see next.

### 2.2. Light.Touch.Matters

*Light.Touch.Matters* was a project that brought together product designers and materials scientists from nine countries with the aim of creating a new generation of smart materials that can sense human touch and respond with luminescence. Applications for these materials were envisioned in the healthcare and well-being sector. Beyond the materials development and design aspects of the project, we also aimed to develop a methodology for designers and materials scientists to work together successfully. The project was conceived in response to an EU call to address the widely acknowledged gap between the commercial product design community who use materials, and materials researchers who develop new materials. This division and lack of communication was perceived by the EU as a barrier to developing new technologies that meet human needs and as a barrier to commercial competitiveness (FP7-NMP).

From the start the *Light.Touch.Matters* team identified two technologies with which to experiment: touch responsive piezoelectric plastics (Khaliq et al. 2017) and flexible organic light emitting diodes (Coenen et al. 2015). The aim of the *Light.Touch.Matters* project was to combine these two technologies to produce a material that would light up when touched, but also had the softness and flexibility to be used in a wide variety of design applications. However, the precise way in which the organic light emitting diodes and piezo plastics were going to be integrated, and what the new hybrid material would look and feel like, were still undecided. The aim of the project was to allow the designers to lead in the development of this *Light.Touch.Matters* hybrid material, which the materials scientists should then try to manufacture.

The project team met approximately every three months over a period of three and a half years. During those meetings, we not only collaborated to create the new material, but we also developed a methodology for working together. This methodology drew on the past experiences of members of the team, but also relied on an extended period of trial and error where we encountered and tried to resolve significant issues with working together. These problems manifested themselves early on in the project when it became clear that there were three communication problems hindering the dialogue between materials researcher and designer:

1. Materials scientists and design partners were using different terms to describe the same material property. When designers specified that they wanted a flexible material, materials researchers were unsure if they wanted something with low hardness or low elastic modulus. Although materials researchers and designers were talking about the same kind of behaviour they were talking about that behaviour using different terms.
2. Materials scientists wanted designers’ materials requirements to be couched in quantitative terms, so that they could begin to incorporate them into the materials specifications that guide their research. For example, when designers talked qualitatively about the new material being very flexible, materials research partners wanted a numerical value for the desired value of elastic modulus.
3. Because of their different disciplinary lenses designers and materials scientists sometimes focused on different kinds of materials qualities that could not be directly translated into the others’ terminology. For example, unlike flexibility, which broadly correlates with elastic modulus and stiffness, designers were concerned with the enchanting, relaxing or tactile qualities of a material. These kinds of human experiences of materials do not directly correspond with a single, measurable physical property like surface roughness, lumen output or thermal effusivity.

We explore these communication problems and some of the methods and tools to deal with them in further detail in an online resource we developed as part of the project (Institute of Making 2016). One of the approaches we took to overcome this problem was to develop a material collection that could be used at workshops to help translate what designers and materials researchers meant when talking about analogous material properties like flexibility and stiffness. In the context of this project, the material collections were intended as ‘boundary objects’ (Star and Griesemer 1989) that would allow consortium partners to share different ways of looking at material properties. The collections needed to allow materials researchers and designers to communicate without designers having to specify numerical values for stiffness, but in a way that would allow materials researchers to extract a quantitative measure to inform research.

**Figure 4.**
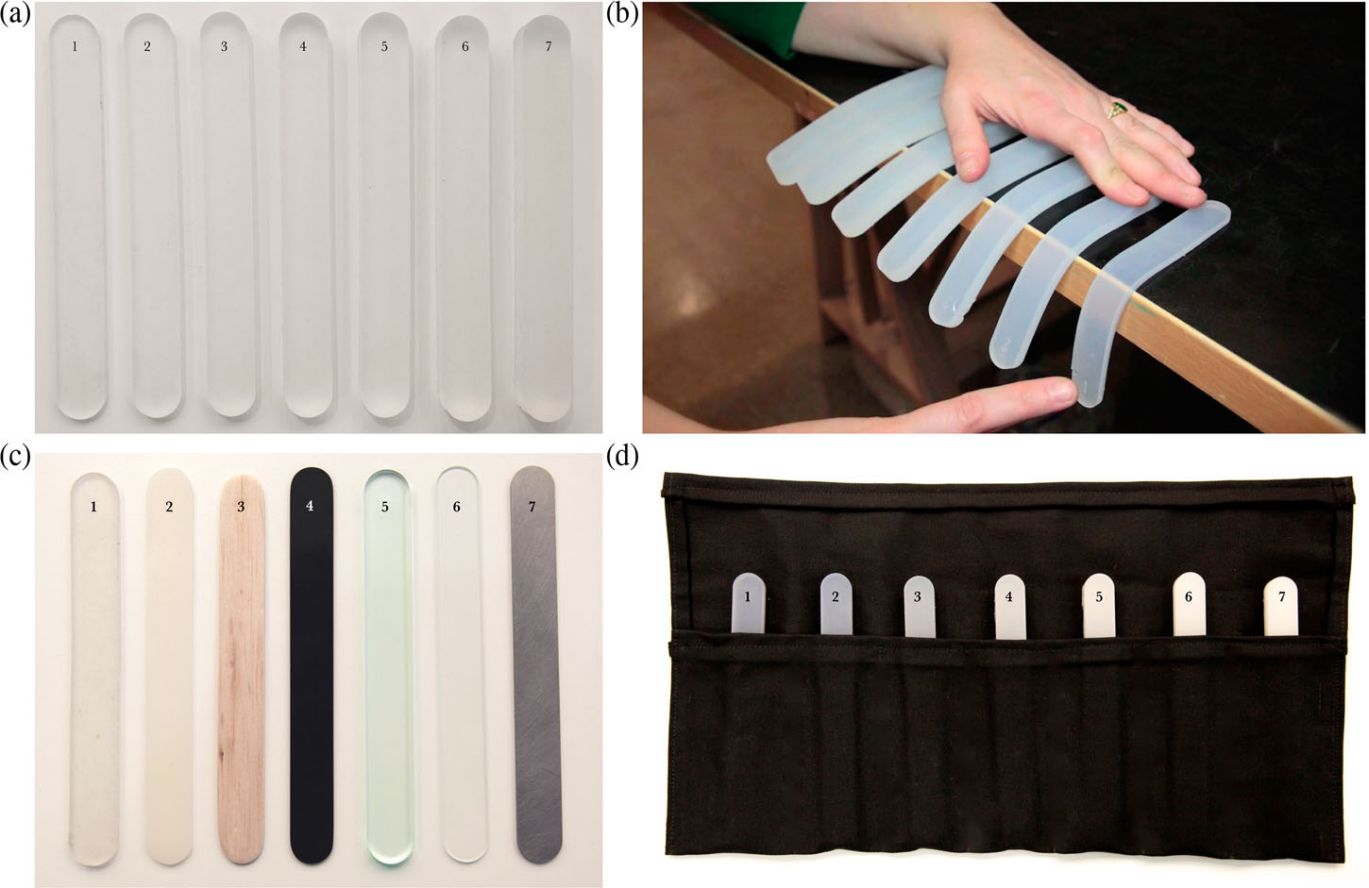
(a,b) A stiffness/flexibility material-object set made from one material (silicone rubber) of varying thickness in 2 mm increments to illustrate that Young’s Modulus remains constant whilst stiffness varies considerably as a result of the form of the object; (c) The seven multi-material samples exploring stiffness were made from a selection of materials with different Young’s Modulus: two different grades of silicone rubber, balsa wood, polystyrene, acrylic, glass and stainless steel. (d) An opacity/transparency material-object set made from one material (silicone rubber) with the same shore hardness, shape and dimensions but different quantities of white dye in a measured gradation.

The outcome was a set of portable ‘tool rolls’ filled with multi-material lollipops (Figure 4). Both the form and the materials were selected to allow us to explore the properties of luminosity, flexibility and tactility in materials: from our observations at the workshops thus far these seemed to be the terms that project partners were struggling most to communicate.

The material collections achieved their primary aim of encouraging useful and grounded discussion between materials scientists and designers. For example, it became apparent that not all designers wanted the maximum light output possible from organic light emitting diodes and that the quality of the light is sometimes more important than the quantity. Not all designers wanted transparency in their silicone rubber top layer, or silicone rubber at all. Discussions about texture began to focus on the kinds of internal texture that might be possible, not just the surface feel of the material. Useful discussions about how we perceive colour and the relationship between opacity, thickness and diffusion of light emerged through play with the tool roll materials. These material collections enabled a different kind of conversation that was not possible simply through verbal description.

However, this use of material collections and making activities did not allow us to tackle one limitation of the *Light.Touch.Matters* project: the design of the project meant that we did not involve end users in the development of the *Light.Touch.Matters* material. This lack of user involvement stemmed in part from the fact that the overarching project structure was market-oriented rather than user-centred, and that the main focus was on the dialogue between materials researchers and designers, as depicted in Figure 2. This project was primarily concerned with speeding up the journey of materials from laboratory to marketplace, so the end users were broadly conceived of as manufacturing companies or brands that would take up the new materials and products. A secondary aim of the project, however, was to develop materials and products that would ‘make consumers feel better, monitor or improve their health or increase comfort’ (LTM website). This work of understanding how materials and product design choices affect user experience was to be led by the design partners. The project brought together numerous different design practice and design research traditions, each with their own methods and with differing opinions as to what extent the input of actual end users was required. One or two of the individual design agencies were interested in participatory or user-centred approaches (Sanders 2002; Sanders and Stappers 2008) and used interviews, focus groups or observational research to inform their design process: exploring the needs of physiotherapists and those undergoing physical rehabilitation and observing how they responded to their designs, for example. Others followed a ‘latent needs’ school of thought, which argues that customers are often not aware of their own needs, or a ‘market-oriented’ or ‘design-driven innovation’ approach (Verganti 2008), which argues that radical innovations should be led by the design firm, not by the user. Proponents of these ‘designer-centred design’ (Koskinen and Battarbee 2003) approaches rely instead on the vision of the designer or on imagined, fictional end users to develop their designs. The structure of the project also meant that neither the material nor the product designs were finalized until the end of the project. This made it difficult to determine who the consumers would be and thus to explore their experiences of the materials being developed.

From the outset our main concern with the smooth running of the *Light.Touch.Matters* project was with the dialogue between scientists and designers around the specification of material properties. The different languages and value systems of materials researchers and design practitioners caused initial communication problems, described as ‘not just a stumbling block but a great big hurdle that we had to get over at the beginning of the project’ (LTM Designers video, 2016). Once this hurdle was overcome by the use of material collections, the project allowed for dialogue between the laboratory and the design studio: something that is still very unusual and yet crucial for the development of materials that meet the sensory and aesthetic requirements of designers. However, one of the main limitations of the *Light.Touch.Matters* project was its lack of attention to the impact that materials choices can have on user experiences. Within the confines of a complex collaborative project, research with users was hindered by the semi-developed nature of the materials (Barati et al. 2015), a market-oriented focus on corporate or imagined end users, and design traditions espousing different approaches to user involvement.

### 2.3. Hands of X

The final case study, *Hands of X*, is an ongoing design-led project that explores the potential for materials choice to impact on people’s everyday experiences of their prosthetic hands. The selection of materials for prosthetic limbs has implications for the wearer beyond function and comfort. Cairns et al. (2014) and Sansoni et al. (2015) have demonstrated that the appearance of a prosthesis affects its acceptance and that improving aesthetic qualities can help to improve the body image and psychological well-being of the wearer. However, relatively little research has been done on the effects of materials choice on the sensory and aesthetic qualities and psychosocial impacts of upper limb prosthetics (Carroll and Fyfe 2004). Prosthetics wearers currently have a relatively limited palette of materials to choose from with regard to the aesthetics of upper limb prosthetics, the most common being skin-like imitation materials such as flesh coloured silicone rubber.

Over the last decade, however, an increasing number of private initiatives have developed that provide wearers with more materials choice in terms of both the aesthetics and function of their prosthetic (The Alternative Limb Project; Open Bionics). Contemporary prosthetics have increasingly used obviously non-skin-like materials such as carbon fibre composites and titanium, prompting prosthetics providers (Pace Rehabilitation 2011) and commentators to posit a general trend away from the ‘uncanny valley’ (Mori 1970) of mimetic, cosmetic limbs towards more creative and conspicuous materials and designs (McDonald 2015). However, the changing material and aesthetic preferences of wearers of prosthetic limbs in the UK are still poorly understood, as are the implications of materials choice on questions of identity, psychological acceptance and body image. Any discussion of ‘trends’ also ignores the fact that high-tech materials and highly decorative bespoke limbs are neither affordable nor appealing to all prosthetics users (Sansoni et al. 2015). *Hands of X* attempted to look beyond simple dualisms of cosmetic and high-tech to examine the individual materials preferences and experiences of wearers, with a view to exploring a nuanced and varied palette of materials, made affordable for prosthetics through digital manufacturing. As depicted in Figure 2, this project connected design researchers directly with users, with the culmination of the project being a prototype of a prosthetics service that offered users a choice of materials.

At an early stage in the project, the design team hypothesized that everyday and familiar materials might be particularly suitable for a bespoke prosthetics service (Pullin 2009). This was informed by work in the field of disability studies (Swain and French 2000) and by Fukasawa and Morrison’s philosophy of the Super Normal: that ‘special is generally less useful than normal, and less rewarding in the long term’, and that ‘special things demand attention for the wrong reasons, interrupting potentially good atmosphere with their awkward presence’ (Morrison and Fukasawa 2006). On the basis of this, the design team conceived of a prosthetics service where wearers can choose from a palette of materials familiar from everyday use, handling and wear, with a view to offering a prosthetic limb that is bespoke but ‘no big deal’.

**Figure 5.**
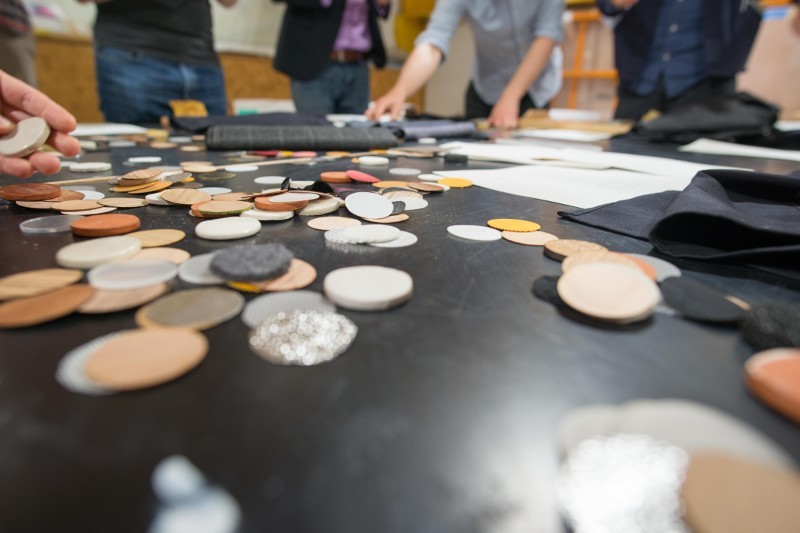
Circular discs of 38 mm in various materials being used as part of a co-design exercise at the *Hands of X* London workshop. Photo courtesy of Chris Natt.

In the course of this project, we used and added to the existing materials library to encourage conversations about materials selection between users of prosthetics and those involved in their design and production. Materials were chosen to be recognizable from contemporary everyday objects and historical prosthetics and included materials as diverse as natural rubber, leather, steel, aluminium, porcelain, concrete, cork, tweed and objects such as brillo pads and moulded leather bicycle saddles. The curated selection also focused on the subtle differences between the same material that had been processed or gained patina in different ways, with representations of different alloys of copper, finishes of stainless steel or ceramic glazes. This curated selection of materials served as the focal point for a series of participatory workshops, co-design exercises and interviews that encouraged participants to focus on the ways in which materials choice could contribute to the prosthetic wearer’s sense of ownership, identity and body image (Figure 5).

It can be particularly hard to talk about materials with end users because they tend to be the backdrop to our lives that often only come to our attention when they fail. It is precisely the quiet ‘humility’ that makes materials invisible that also makes them very powerful in influencing our behaviour and experiences (Miller 1987). In this project, the materials library offered a creative and non-verbal way to talk to wearers about materials for prosthetics that would not otherwise have been possible.

**Figure 6.**
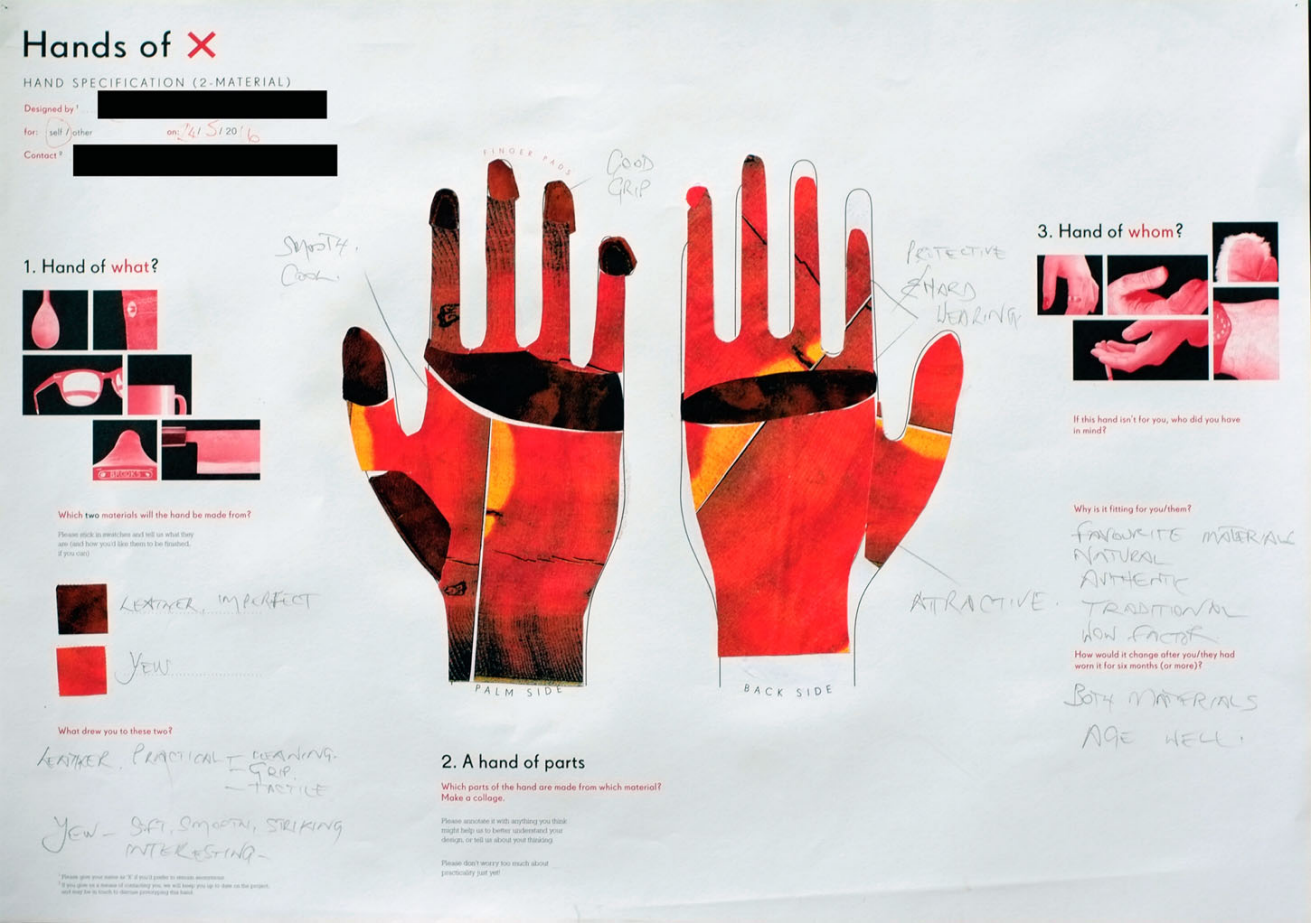
One of the 78 designs of ‘hands in two materials’.

The participatory workshops and interviews were primarily aimed at giving us a better understanding of the material and aesthetic preferences of a small group of prosthetics wearers, as well as engaging a wider community of artists and designers in the design of prosthetics. The main outcome of the workshops was a series of 78 designs of ‘hands in 2 materials’ conceived by wearers, prosthetics, engineers and designers (Figure 6).

The disciplinary differences of the research team became evident after the participatory workshop activities. Coming from an engineering and social science research perspective, the materials researchers were interested in understanding how the experiences of individuals who took part in the workshops related to the needs of a broader prosthetic wearing community. The materials researchers saw the 78 designs from the workshops as a source of qualitative data that could be analysed to explore participants’ materials preferences. For example, looking at the group as a whole there was as much discussion amongst participants of exotic, luxurious or precious materials as familiar or ‘super normal’ materials. Following the workshops, the materials researchers, therefore, saw the need for systematic research to gather robust data from a larger sample of people in order to develop a user-centred palette of materials that would help healthcare providers and prosthetics manufacturers select materials for prosthetics.

**Figure 7.**
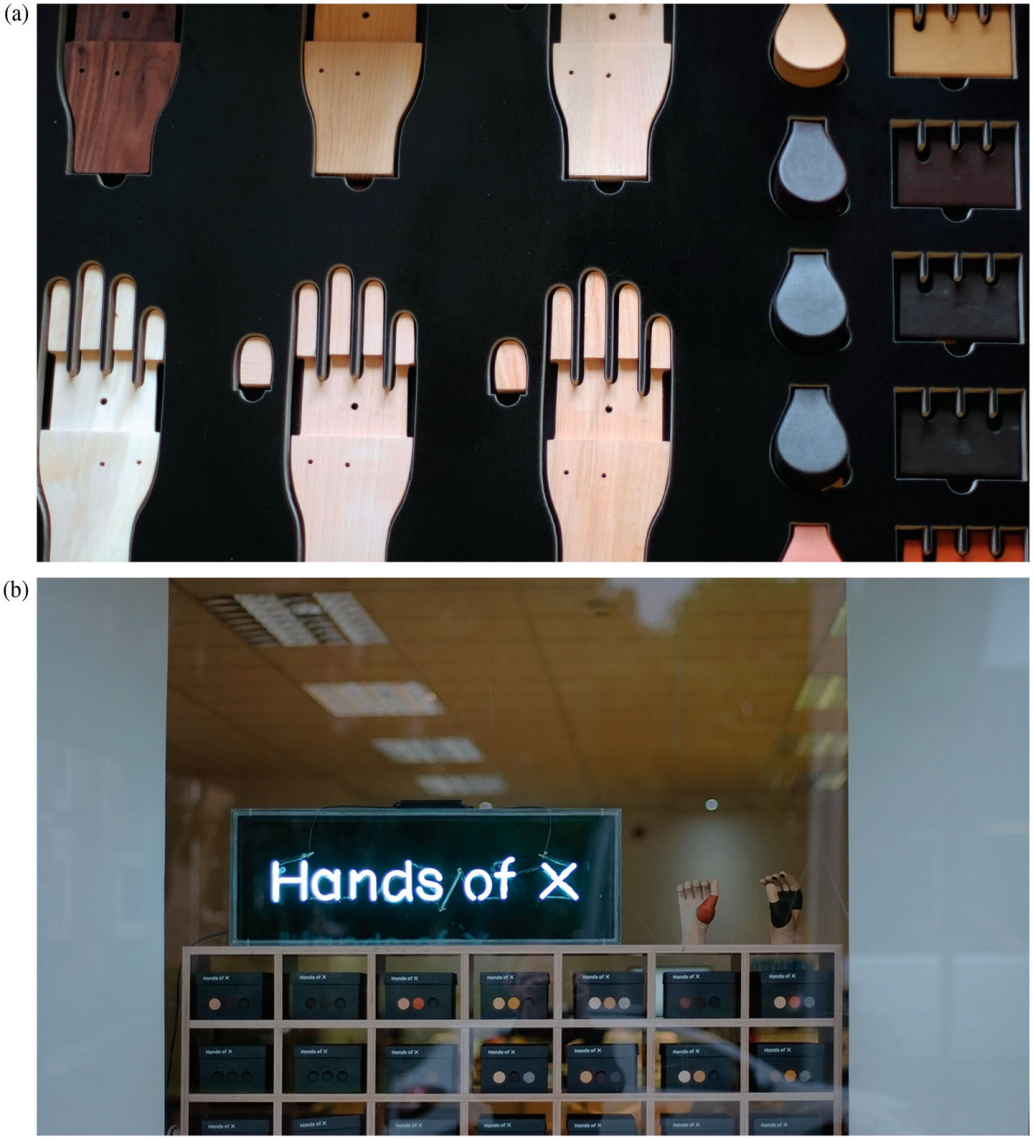
Prototype bespoke prosthetics service developed by the designers.

The instinct of the design researchers, on the other hand, was to remain committed to the original vision of prosthetics that were understated and ‘no big deal’, concentrating in on those wearers that the service spoke to rather than trying to offer ‘something for everyone’. For them, the materials palette should be informed by the results of the participatory workshops but could be filtered by their design agenda. This difference in approach was due to the different research traditions of members of the *Hands of X* team. Coming from an arts and design tradition, the main focus for the design researchers was on provocation and inspiration; research as a way of shifting cultural, political and aesthetic expectations of assistive technologies rather than as a way of collecting data or solving a problem. The resulting prototype of the service, carried out in a London high street shop, was indeed extremely impressive and well received by users, as well as achieving the ambitions of the designers (Figure 7).

These diverging approaches generated interesting discussions about the nature and priorities of research across these different disciplines. They also stemmed in part from a terminological misunderstanding. In developing the project together, we stated that it would be user-centred and would employ a co-design approach. However, the fundamental differences in what different members of team perceived co-design to be only became apparent later in the project. The materials researchers understood this to mean a participatory approach where users inform and become part of the creative process from start to finish (Sanders and Stappers 2008). For the design researchers, this kind of ‘participationism perspective’ risked reducing the design process to ‘a polite conversation’ around a table with stakeholders and threatened to transform design experts into ‘administrative actors with no specific contributions to bring – other than aiding the process with their post-its’ (Manzini and Coad 2015). The subtle differences between different kinds of user involvement and dialogue, and the resulting implications for the expertise of the designer (Gaver, Dunne, and Pacenti 1999), are well understood and still much-debated within the design community (Steen 2011), but only became apparent to the materials researchers in the course of the project. This demonstrates that the work involved in developing a common language between the disciplines of materials research and design is still ongoing. Whilst the materials collection played a crucial in the *Hands of X* project as a design tool it did not solve all our methodological differences and terminological misunderstandings.

## 3. Discussion and conclusions

Each of the interdisciplinary projects discussed here had its own rewards, complications and limitations. Although the aims of these projects and the stakeholders involved were very different, all three demonstrate difficulties involved in doing interdisciplinary work. Working between disciplines often seems like the only way to address complex problems, but there is always a risk, as we found frequently, that collaborating partners will be unable to resolve conflicts in epistemologies, value systems and methodological traditions (Bracken and Oughton 2006; Bell, Marzano, and Carss 2005).

The issues at stake should not be underestimated: using another discipline’s definitions is not just about translation; there are value judgments intrinsic to disciplines that are embedded in the language they use. Those values are often linked to judgments of excellence: of what makes an excellent project, an excellent product or an excellent piece of work. Asking materials scientists to adapt to using a less quantitative language of softness, for instance, based on touch perception rather than a number measured by a machine, is a significant request. People who have spent their whole careers working with one set of materials parameters have done so for a reason, because they find it an effective way to make progress in their work. Asking them to change this for an interdisciplinary project is hard. Conversely, materials scientists requesting numbers about the required softness from designers so that they can go back to the laboratory and create it is antithetical to the way many designers work.

A great deal has been written about the ‘paradigm wars’ and difficulties of collaborative working for researchers that espouse largely qualitative or quantitative research methods. In our experience, as described in these case studies, tensions also arise as a result of the primacy that each partner gives to creativity, the development of new knowledge and to solving societal problems. In the *Hands of X* project, the main focus was on a creative, design-led approach, in which materials research was secondary, which created tension. The *PhysFeel* project employed traditional social science research methods but was not clear enough about the end use of the new knowledge, opening up confusion about the applicability of the results in a design context. In the *Light.Touch.Matters* project, the primary aim was to use a design-led materials development methodology to create new materials, but it did not explicitly include end users in the process, leading to disagreement about its societal relevance.

In the particular disciplinary triangle (Figure 2) between materials researchers, designers and users, collaboration is further complicated by differences between academic and professional practice: in acceptable timescales for research, which may vary from weeks to years; and the motivating factors of the different parties, which might vary from research papers to exhibitions or marketable products. The impetus to develop a successful physical product or to have a direct impact on end users can be important driving forces for interdisciplinary collaborations. The power of the ‘research object’ product can sometimes overshadow ‘epistemic objects’: those aspects of a research project with no concrete, physical instantiation (Rheinberger 1997).

Although materials libraries are not a panacea for all the problems associated with interdisciplinary working, the projects discussed here show a variety of ways in which material collections can act as both ‘things to think through’ (Henare, Holbraad, and Wastell 2007) and as tools for communication. In the *Light.Touch.Matters* project, material collections were successfully used to overcome disciplinary language barriers between design professionals and materials researchers in order to develop their working relationship. In the *PhysFeel* project, they were used to systematically gather data about how participants might use material properties to communicate affect. In the *Hands of X* project, they were used as provocations to draw attention to the idea that materials used in upper limb prosthetics have an impact on identity, ownership and body image. These three examples show that material collections can play several roles: enabling interdisciplinary translation and creating relationships between multidisciplinary groups of people; provoking dialogue with potential users of a material or service; and allowing us to ask research questions they we could not otherwise contemplate.

Crucially, each of these projects required a different specially made or curated collection of materials that was developed either with or in response to the project team and participants. Thus materials libraries as tools for interdisciplinary research are not static and unmediated collections of objects, but a series of interventions. These interventions deliberately draw on the theories and methods of the physical scientists, artists and designers and social scientists that use them. Informed by materials science approaches, material collections explore the measurable physical, chemical and mechanical properties of materials as well as their perceived sensory and aesthetic attributes. Drawing on the ‘cultural probes’ (Gaver, Dunne, and Pacenti 1999) tradition in design research, material-objects can also act as provocations to encourage discussion, communication, play and sometimes discomfort and disagreement rather than consensus. Informed by material culture ideas of ‘cognitive scaffolds’, these allow us to ‘think things that we could not otherwise contemplate’ (Day 2004; Pedersen 2007): they enable us to draw people’s attention to normally humble materials to explore how they influence our behaviour and experiences. Drawing on these different disciplinary approaches allows us to situate the materials library between disciplines as a shared research tool: materials libraries are deliberately distinct from discipline-specific research tools (graphs, datasheets and product concepts) as well as the invisible and shared material culture (powerpoint presentations; ‘descriptions of work’) that gives interdisciplinary work its infrastructure and solidity (Star 1999).

## Footnotes

1 Whilst this schematic necessarily simplifies the complexity of the experimental entanglements between these different players and the often-fierce debates within each discipline (Fitzgerald and Callard 2015), we found it useful as a discursive tool to help us think broadly about how different disciplinary traditions and epistemologies have influenced our use of the materials library.
